# Clinical relevance of extracellular vesicles in hematological neoplasms: from liquid biopsy to cell biopsy

**DOI:** 10.1038/s41375-020-01104-1

**Published:** 2020-12-09

**Authors:** Stefania Trino, Daniela Lamorte, Antonella Caivano, Luciana De Luca, Alessandro Sgambato, Ilaria Laurenzana

**Affiliations:** 1 Laboratory of Preclinical and Translational Research, Centro di Riferimento Oncologico della Basilicata (IRCCS-CROB), Rionero in Vulture, PZ Italy; 2 Laboratory of Clinical Research and Advanced Diagnostics, Centro di Riferimento Oncologico della Basilicata (IRCCS-CROB), Rionero in Vulture, PZ Italy; 3 Scientific Direction, Centro di Riferimento Oncologico della Basilicata (IRCCS-CROB), Rionero in Vulture, PZ Italy

**Keywords:** Cancer, Haematological cancer

## Abstract

In the era of precision medicine, liquid biopsy is becoming increasingly important in oncology. It consists in the isolation and analysis of tumor-derived biomarkers, including extracellular vesicles (EVs), in body fluids. EVs are lipid bilayer-enclosed particles, heterogeneous in size and molecular composition, released from both normal and neoplastic cells. In tumor context, EVs are valuable carriers of cancer information; in fact, their amount, phenotype and molecular cargo, including proteins, lipids, metabolites and nucleic acids, mirror nature and origin of parental cells rendering EVs appealing candidates as novel biomarkers. Translation of these new potential diagnostic tools into clinical practice could deeply revolutionize the cancer field mainly for solid tumors but for hematological neoplasms, too.

## Introduction

In the last decades, precision medicine has emerged as a powerful clinical strategy in oncology to tailor therapies for an individual patient, thus providing a significant improvement in clinical evolution and outcome of cancer patients [1].

To date, tissue biopsy represents the current gold standard for cancer diagnostics, but the acquisition of tumor tissue presents several complications, such as being expensive, invasive, and negatively affected by tumor heterogeneity, providing a single snapshot in time and at risk of potential complications which may require hospitalization [2, 3].

Liquid biopsy has emerged as an innovative and noninvasive approach to diagnose and monitor patients allowing to overcome the limitations of conventional biopsy. It consists in the isolation and analysis of tumor-derived materials in bodily fluids, such as blood, urine, and saliva, and offers interesting opportunities for the identification of novel diagnostic and prognostic biomarkers [1]. In particular, this approach allows the detection of circulating tumor cells, cell-free DNA, and extracellular vesicles (EVs), deriving from primary and metastatic sites. These tumor-derived materials represent a source of genomic and proteomic information potentially useful for early diagnosis, risk stratification, disease monitoring, and personalized treatment selection for cancer patients [1, 3].

The term “extracellular vesicles” is used to describe particles delimited by a lipid bilayer that are heterogeneous in terms of size, biogenesis, and composition; they are unable to replicate and are naturally released from cells in both physiological or pathological situations [4]. For many years, EVs have been classified into exosomes, ectosomes (microparticles (MPs) and microvesicles (MVs)), apoptotic bodies [5], and large oncosomes [6]. In “Minimal information for studies of extracellular vesicles 2018” update, the terms “small EVs” (<200 nm) and “medium/large EVs” (>200 nm) have been proposed to replace “exosomes” and “ectosomes”, respectively [4].

It is well established that biogenesis processes, together with environmental conditions, epigenetic changes, and developmental stages, are crucial moments in which EVs receive, from their parental cells, bioactive molecules, such as proteins, lipids, metabolites, and nucleic acids (coding/noncoding RNAs, genomic and mitochondrial DNA) forming their cargo [6].

**Fig. 1:**
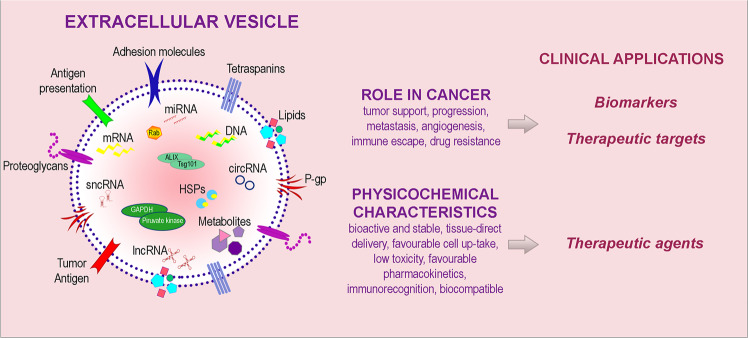
Role of EV repertoire and potential clinical applications in cancer. EV cargo includes bioactive molecules on EV surface (adhesion molecules, tetraspanins, molecules involved in antigen presentation, proteoglycans, lipids, P-glycoprotein P-gp and tumor specific antigens) and molecular content (DNA, mRNA, microRNA, long noncoding RNA, short noncoding RNA, circular RNA, metabolites, heat shock proteins, enzymes). EVs and their components play multiple roles in cancer and have peculiar physicochemical characteristics, thus holding a potential clinical utility as biomarkers as well as therapeutic targets and agents.

Nowadays, the real goal in cancer diagnostic area is the discovery of new biomarkers able to provide tumor information before treatment and to improve therapeutic plan and monitoring. In this context, EVs and their cargo could be used as biomarkers offering a multicomponent diagnostic window; moreover, EV key role in tumor growth, renders them a potential target for novel therapeutic strategies. Finally, EV functional properties also favor their use as therapeutic vehicles [1, 7–9] (Fig. 1).

In this review, we provide an up-to-date account of EVs as novel “multiomic shell” biomarkers, potentially suitable in a future clinical practice for detection, prediction of response/resistance to treatment and minimal/measurable residual disease (MRD) monitoring in lymphoid and myeloid neoplasms.

### EV clinical applications in hematological malignancies

Hematological malignancies (HMs) are clinically and biologically heterogeneous diseases. In terms of clinical heterogeneity, affected patients may require different therapies to which they could respond or develop resistance. Similarly, biological heterogeneity includes genetic, molecular, morphologic, and phenotypic variability [10]. Moreover, HMs could be considered “dynamic pathologies” characterized by the accumulation of genetic alterations and by the co-existence of competing cellular clones. In particular, the genetic landscape is constantly reshaped during disease progression [11, 12]. For these reasons, a prompt management of HMs, including correct diagnosis, risk stratification and continuous disease monitoring, is important to establish appropriate therapies and to assess prognosis. In this context, an important goal in clinical practice is the identification of novel biomarkers, sensitive and representative of tumor heterogeneity, which could improve HM diagnosis and disease monitoring.

Actually, diagnostics of HMs, due to their complexity, is based on an integrated approach incorporating clinical, morphological, immunophenotypical, and genetic data, altogether finalized for guiding clinical management.

EVs reflect physiological or pathological states of origin cells with their specific content profile, and thus could be considered as “cell biopsies” representing tumor information vehicles [8]. Furthermore, EVs could serve as surrogate of disease presence and their evaluation, specifically in peripheral blood (PB), could provide an early and highly sensitive method for cancer detection and monitoring of disease progression [13].

To date, more than thousand separation methods have been developed and evaluated for EV recovery rate, purity, and processing time [13]. Typical approaches are based on different isolation principles such as size, density, surface charge, hydrophilic interactions with solvents and affinity for biological targets (e.g., ultracentrifugation, density gradient, size-exclusion and ion-exchange chromatography, sucrose density gradient, polymer-based precipitation, and immunoisolation) [4, 14]. Each method shows advantages/disadvantages and diverse specificity/efficiency of purification. Consequently, given EV heterogeneity, the use of complementary methods is recommended to provide a better particle isolation [4].

EVs, being a surrogate of cells, can be analyzed for the same cell-based parameters, such as count, phenotyping and molecular content, routinely evaluated in cell-based HM diagnostics, thus becoming powerful biomarkers in tumors. EV abundance and stability in blood render their analysis more advantageous compared to cell-based liquid biopsy strategies.

Upon suspect of HM, generally, complete blood cell count is the first step in the diagnosis and it is performed by an automated cell counter which reveals the presence/quantification of an abnormal cell count. Similarly, EV measure, easily and quickly performed by nanoparticle tracking analysis which quantifies small particles ranging from 50 nm to 1 µm [14], and by cytofluorimetric analysis [15], could help to identify a pathological state.

HM diagnostic methods include cytomorphology and immunephenotyping whose combination is useful to discriminate between normal and abnormal cells. In particular, multiparameter flow cytometry (MFC) immunophenotyping provides accurate assessment of specific multiple markers expression, even when malignant cells are present at low frequencies [16], and its sensitivity and multiparametric ability allow to analyze approximately hundred molecules per cells [17].

At the same way, EVs can be morphologically analyzed by different microscopy techniques, including scanning or transmission electron microscopy, cryo-electron microscopy, atomic force, and super-resolution microscopes [4], whose application highlighted shape differences between tumor- and healthy-derived EVs. At phenotypic level, FC can also stratify EV population according to specific antigen expression levels [18]. Moreover, although conventional FC lacks the sensitivity to accurately measure EVs, it remains the fastest method to identify EVs in clinical samples allowing a multiplex fluorescent detection. Moreover, to overcome sensitivity difficulties, high-resolution and nano-flow cytometry, and fluorescence-activated cell sorting have been developed to better count, sort and investigate the phenotype of medium/large EVs.

Protein analysis is an integral part of diagnosis and monitoring of different HMs. For example, M-protein detection and quantification are commonly evaluated in monoclonal gammopathies by electrophoretic and immunochemical methods [19].

Concerning EV protein cargo, conventional western blotting and enzyme-linked immunosorbent assays can be applied for protein analysis. However, these methods, quantifying targeted proteins in a relatively small scale, are less suitable for clinical use, especially for studies involving large patient cohorts or quantification of rare markers [20]. Interestingly, Hoshino et al. developed a novel method based on mass spectrometry analysis able to identify tumor-associated EV protein signatures with high sensitivity and specificity, indicating them as useful liquid biopsy tool to support cancer diagnosis and treatment response [21].

In cell-based HM diagnostics, molecular techniques can specifically detect targeted abnormalities known to have a significant clinical impact; for example qRT-PCR measurement of BCR-ABL1 transcript levels, in chronic myeloid leukemia (CML), allows to detect as few as one malignant cell in 10 × 10^4^ nonmalignant ones [22] and NPM1 quantitative assessment in acute myeloid leukemia (AML) which median sensitivity of detection is 1 × 10^−5^ [23]. Interestingly, both transcripts have been respectively found in CML and AML derived-EVs [24, 25]. Regarding EV molecular assessment, different studies analyzed the abundance and the stochiometric presence of RNA in EVs revealing that any given transcript is present on average lower than one per vesicle/particle in an EV sample [26–28]. However, although these studies could indicate that RNA detection is characterized by a low analytical sensitivity that might not allow to establish EV nucleic acids as biomarkers, several considerations should be made. For instance, it is possible that larger EVs may carry significant numbers of miRNA molecules and that other classes of RNA may be packaged differently than miRNAs [26]. In addition, a limiting factor in stochiometric analysis could be represented by the applied methodology. In fact, despite qRT-PCR is a highly sensitive method (detection limit of pg-fg), it does not measure total nucleic acid amount and is only suitable for detection and quantification of known and specific sequences [14, 29]. Moreover, different RNA sequences can greatly differ in abundance, so it is difficult to accurately extrapolate indications on the total amount of EV RNA by assessing one specific RNA. On the other hand, next generation sequencing (NGS), which is now widely applied for EV-derived nucleic acid analysis, allows the simultaneous evaluation and comparison of multiple samples and the discovery of novel RNAs, representing a suitable technique for unbiased identification of EV RNA content [30].

Furthermore, another highly sensitive promising approach for small pathological clones detection is based on droplet digital PCR. Advantageously, absolute nucleic acid quantification can detect low amounts of target (up to 0.001% mutated allele frequency) [31] and therefore it is suitable for EV nucleic acid analysis, as demonstrated by detection of BCR-ABL1 transcript in CML-derived EVs [32].

Currently, the scientific community shows an increasing interest in developing standardized methods in EV field. Of note, the National Institutes of Health Common Fund launched a new program, the Extracellular RNA Communication Consortium, to establish whether extracellular RNAs (exRNA) and their carriers, including EVs, can be utilized for clinical applications, aiming to develop robust and reproducible methods for EV/exRNA isolation and analysis [33]. In particular, in a recent study, the Consortium reported the high intra- and inter-lab reproducibility of commonly used small RNAseq methods on EV RNA [34].

Regarding MRD monitoring in HMs, MFC and molecular analysis are the gold standard cell-based methods. The first technique, which can reach a sensitivity of 10^−3^ to 10^−5^ of leukemic blasts [35], is not recommended on PB due to the lower presence of leukemic cells [36]; instead, qRT-PCR, which has a sensitivity of 10^−4^ to 10^−6^, relies on specific genetic or molecular targets and, thus, is applicable to fewer patients [35]. In this context, thanks to their abundance in biological fluids, specifically in blood, tumor-derived EVs could overcome the deficiency of residual malignant cells making PB sampling suitable for MRD monitoring.

For this reason, PB-EV-based liquid biopsy could offer a noninvasive, fast, and pain-free diagnostic tool. This approach could become complementary to current painful bone marrow (BM) biopsies, which expose patients to repetitive BM punctures and imply the risk of not representative sampling due to tumor heterogeneity [19].

Moreover, by homogenously circulating in various body fluids, EVs can capture the entire cancer heterogeneity; thus, they have a representative and global profile allowing to monitor tumor changes in real-time. It is also possible that EVs could be identified even when the presence of malignant cells is below the detection threshold of currently used techniques [36].

To date, given that current classification of many HM relies on consolidating parameters, EV analysis should not replace actual cell-based BM diagnostic methods, but EV biomarkers could be used to improve current risk stratification. Notably, since PB is the mirror of BM and given the great potential of EVs, these last could, futuristically, complement or, even, replace the invasive and painful BM sampling. To these purposes, standardization advances, passing from EV isolation to analysis, and studies on a larger cohort of patients are needed to definitively assess EV suitability as biomarkers in HMs.

Currently, a key aspect in defining translational relevance of EVs as biomarkers is their clinical specificity. In this context, several studies reported that the same EV components, such as proteins or RNA cargo, are altered in different types of tumors. However, it is important to point out that these molecules, likewise cell-derived biomarkers, are involved in critical and common processes known to be impaired in tumors, such as cell survival, proliferation, and differentiation. Indeed, it is reasonable that the same molecules could be detected in multiple pathological conditions. For example, miR-155, that plays a critical role in the pathogenesis of several HMs, was found deregulated in serum EVs from these neoplasms [37]. However, up and downregulation of these markers could be influenced by several factors, such as cellular context and biological interactions in different tumor types.

Another consideration is relative to the finding that pathological states determine an increase of EV release. In particular, EVs are released from cancer cells at a higher rate than from healthy ones and they are enriched in tumor signature molecules [38]. This feature could indicate that, at least in patients at diagnosis in which tumor burden is reasonably elevated, it could not be strictly required to separate EVs released from malignant cells. However, mainly during disease monitoring, it may be difficult to discriminate EVs released by residual tumor cells and those released by microenvironment. In addition, it is well known that sustained exposure to the inflammatory process can contribute to the initiation, promotion, growth, and invasion of tumors by providing bioactive inflammation-related molecules, including EVs, that can infiltrate tumor microenvironment [39]. Generally, different pathological conditions, such as acute or chronic inflammation, diabetes, and kidney injury, that concomitantly occur with tumor, could determine EV release from nonneoplastic cells, causing non-specific biomarker modifications.

Importantly, in order to define EVs as novel tumor biomarkers, it would be appropriate to evaluate the association and combination of multiple components. In particular, for each disease, studies on large cohorts of subjects, some of which are currently ongoing, should be carried out to analyze EV count, phenotype and molecular content, possibly identifying a combined panel resulting from the following parameters: (1) number, (2) surface antigens, and (3) RNA/DNA/protein content. Thus, we believe that a detailed and deep characterization of all these parameters in a simultaneous manner, should be useful to discriminate EVs deriving from a specific disease.

Nowadays, to meet the need to better analyze EVs, novel techniques are being developed. In particular, the enrichment of cancer specific-EVs from blood has been obtained through immunomagnetic or immunoaffinity approaches, by using antibody-coated beads or antigen-specific biomarkers, respectively. These novel approaches may guarantee an increased purification efficiency and a targeting of specific EV population. In addition, by high-resolution flow cytometry-based methods, it has been demonstrated the possibility to sort EVs derived from immune and tumor cells with fidelities of 78 and 99%, respectively [40]. Thus, it is reasonable to think that the implementation and, possibly, the combination of these technologies could enables, in the near future, multiparametric characterization and sorting of individual EVs in a manner that is currently not feasible by conventional bulk approaches.

The following sections report evidences underlining potential roles of EVs and their content in HM management, proposing them as a novel prospective tool for diagnosis, prognostication and monitoring of lymphoid and myeloid malignancies (Table 1).

**Table 1:** Serum and plasma EVs and their content as potential biomarkers in hematological neoplasms.

Disease	Biofluids	EV isolation methods	EV analysis methods	EV biomarkers	Alteration/impact	Ref
MGUS SMM MM	Serum	Ultracentrifugation	FC	Number	Higher in MM at diagnosis vs. HDs/MGUS	[15]
	Plasma	Ultracentrifugation + DSG	AFM, CGN + SPR			[44]
		Centrifugation + immunolabelling	FC, SEM		Higher in MM cohort (diagnosis, partial remission, complete remission, relapse) vs. HDs	[45]
	Serum	Ultracentrifugation	FC	CD38	Higher in MM at diagnosis vs. HDs; higher in III vs. I–II ISS stage	[15]
	Plasma	Differential centrifugation	DLS, FC, HPLC	CD38, CD203a (PC-1), CD73, CD157, CD39	Higher in MM at diagnosis vs. MGUS/SMM	[46]
				CD203a (PC-1), CD73	Higher in I–II vs. III ISS stage	
				CD38, CD157	Higher in MM with increased PC level	
		Centrifugation + immunolabelling	FC, SEM	CD138	Higher in MM at diagnosis vs. HDs; increased during progression; decreased in response to therapy	[45]
		Ultracentrifugation	FC		Higher in MM at diagnosis vs. HDs; increased during progression	[47]
		Centrifugation	FC, TEM		Higher in MM at diagnosis vs. HDs; Higher in MM with bone lesion	[48]
				Co-expression CD38/CD138	Higher in MM at diagnosis vs. HDs	
			FC	CD138^-^/P-gp^+^/PS^+^/CD34^+^	Higher in aggressive/non responsive MM	[49]
		Exosome precipitation kit	ELISA	CD163 CD206	Higher in MM at diagnosis vs. remission/MGUS and HDs	[51]
				CD163	Higher in MM at diagnosis vs. relapse	
	Serum	Exclusion chromatography	Immune-blotting	CD44	Higher in MM at diagnosis vs. HDs	[52]
		Precipitation with polyethylene glycol and Protamine + centrifugation	FC	CD146	Indicative of an increased risk of GVHD	[53]
				CD31, CD140-α	Indicative of a lower risk of GVHD	
		Differential centrifugation + exosome precipitation kit	qRT-PCR	let-7b, let-7e, miR-106a, miR-106b, miR-155, miR-16, miR-17, miR-18a, miR-20a	Low expression predictive for PFS	[54]
				let-7b, miR-18a	Low expression predictive for OS	
		Ultracentrifugation	AFM, TEM, qRT-PCR	miR-155	Lower in MM at diagnosis vs. HDs	[37]
		Exosome precipitation kit	qRT-PCR	let-7c-5p, let-7d-5p, miR-185‐5p,		[55]
				miR-20a‐5p, miR‐103a‐3p, miR‐425‐5p	Lower in MM at diagnosis/SMM vs. HDs	
				miR-4741 miR-4505	Higher in MM at diagnosis vs. HDs; higher in MM at diagnosis vs. SMM/HDs	
				let-7c-5p, miR-20a‐5p, miR‐103a‐3p, miR‐140‐3p, miR‐185‐5p	Lower in MM at diagnosis vs. SMM	
	Plasma	Ultracentrifugation	qRT-PCR	miR-129-5p	Higher in MM at diagnosis vs. SMM	[56]
	Serum	Centrifugation		miR-16-5p, miR-15a-5p, miR-20a-5p, miR-17-5p	Lower in bortezomib-resistant MM	[57]
		Exosome isolation kit	qRT-PCR	lncRNA PRINS	Lower in MM at diagnosis vs. MGUS/ HDs	[58]
HL, NHL	Serum	Ultracentrifugation	FC	Number	Higher in HL/NHL at diagnosis vs. HDs	[15]
	Plasma	SEC				[62]
	Serum	Ultracentrifugation		CD30	Higher in HL at diagnosis vs. HDs	[15]
				CD19	Higher in NHL at diagnosis vs. HDs	
			ELISA	CD20	Reduction of rituximab efficacy	[63]
		Differential centrifugation	Acetylcholinesterase activity, qRT-PCR	BCL-6 mRNA	Higher in DLBCL vs. FL vs. HDs; high death rate in patients with first relapse and nonresponder	[64]
				C-MYC mRNA	Expression in poor PFS FL	
				AKT	Worse PFS in therapy responder patients	
				BCL-XL	High death rate in FL patients after treatment	
		SEC	FC	miR-24-3p, miR-127-3p, miR-21-5p, miR-155-5p, let-7a-5p	Higher in relapsed HL vs. HDs	[62]
		Exosome precipitation kit	NGS	miR-99a-5p and miR-125b-5p	Higher in chemoresistant vs. chemosensitive DLBCL; higher in shorter PFS	[66]
CLL	Serum	Ultracentrifugation	FC	Number	Higher in CLL at diagnosis vs. HDs	[15]
	Plasma		NTA			[69]
	Serum		FC			[70]
					Higher in advanced Rai clinical stage; higher in patients with shorter time to treatment and shorter OS	
	Plasma		NTA		Decreased in ibrutinib responder patients	[69]
	Serum		FC	CD19	Higher in CLL at diagnosis vs. HDs	[15]
				CD19, CD20, CD37	Higher in advanced Rai clinical stage	[70]
	Plasma	Differential centrifugation	FC, TEM	CD52	Higher in untreated CLL vs. HDs; high levels in disease progression	[71]
		Ultracentrifugation + floatation on Optiprep cushion	EM, NTA, FC, WB, CM	S100-A9 protein	Present in progression state vs. diagnosis/indolent state	[72]
		Ultracentrifugation	qRT-PCR	miR-150, miR-155, miR-29a-c	Higher in CLL at diagnosis vs. HDs	[69]
	Serum		AFM, TEM, qRT-PCR	miR-155		[37]
	Plasma	Ultracentrifugation + exosome precipitation kit	NTA, qRT-PC, WB	miRNA signature	Higher in therapy-resistant CLL vs. RS	[68]
WM	Serum	Ultracentrifugation	FC	Number CD19	Higher in WM at diagnosis vs. HD; higher in high vs. intermediate/low IPSS	[15]
	Plasma		EM, NTA, qRT-PCR	miR-192-5p, miR-320b, miR-21-5p	Increased level with the disease stage	[75]
				let-7d	Decreased level with the disease stage	
	Serum		AFM, TEM, qRT-PCR	miR-155	Higher in WM at diagnosis vs. HDs; higher in high/intermediate IPSS	[37]
AML	Serum	Ultracentrifugation	FC	Number	Higher in AML at diagnosis vs. HDs	[15]
				CD13	Higher in AML at diagnosis vs. HDs	
		SEC + ultracentrifugation	WB, FC	Number	Higher in AML at diagnosis vs. HDs	[82]
				CD34, CD117, CD33	Higher in AML at diagnosis vs. HDs	
	Plasma		TEM, NTA, FC, ELISA	Total proteins, TGFb-1	Higher in AML at diagnosis vs. HDs; decreased after induction CT; increased during consolidation CT; normalized in long-term CT; reflect response to therapy; related to blast presence in BM	[83]
		Ultracentrifugation	NTA, qRT-PCR, TEM	FLT3-ITD, NPM1	Reflect mutational status of patient blasts	[84]
	Serum		AFM, TEM, qRT-PCR	miR-155	Higher level in AML at diagnosis vs. HDs	[37]
		Exosome isolation kit	qRT-PCR, WB	miR-10b	Higher level in AML at diagnosis vs. HDs; Higher in different AML subtypes; Higher in AML with shorter OS and DFS	[86]
		Exosome precipitation Kit	EM, NTA, WB, qRT-PCR	miR-125b	Higher in patients with elevated risk of relapse and overall death	[87]
	Plasma	Differential centrifugation	FC, qRT-PCR, TEM	miR-150, miR-155, miR-1246	Higher in AML at diagnosis vs. HDs	[81]
	Serum	Ultracentrifugation	NTA, TEM, WB, NGS, GeneScan based fragment-length analysis	dsDNA (NPM1, FLT3, WT1, GATA2, ETV6, ZRSR2, NOTCH1, NRAS, KIT, PHF6)	Reflect mutational status of patient blasts	[85]
CML	Serum	Ultracentrifugation	FC	Number CD13	Higher in CML at diagnosis vs. HDs	[15]
	Plasma	Total exosome isolation kit	Digital PCR	BCR-ABL1	Expression in chronic/blast/accelerated phases; reduction during TKI treatment	[32]
	Serum	SEC + exosome precipitation solution	Nested PCR			[24]
	PB	Total exosome isolation kit	Low-density array	miR-140-3p	Higher in CML with musculoskeletal pain	[95]
Ph^-^MPNs	Plasma	Not applied	FC, MP-activity, ELISA	Number, platelet-/erythrocyte- MPs, MP procoagulant activity	Higher in MPN at diagnosis vs. HDs; higher risk of thrombosis	[96]
			MP-activity assay	Number, platelet-/erythrocyte- MPs, MP procoagulant activity	Higher in MPN at diagnosis vs. HDs; higher risk of thrombosis	[97]
			FC, allele-specific PCR	Number, red blood cell-/endothelial-MPs	Higher in MPN at diagnosis vs. HDs; higher in thrombotic complications	[98]
		Centrifugation	FC, NGS, thrombin generation assay	Tissue factor^+^ MPs, MP procoagulant activity	Higher in MPN at diagnosis vs. HDs;higher in thrombotic complications	[99]
			FC	Number, platelet-/endothelial-MPs	Decreased after therapy	[100]
		Not applied	FC, MP-activity assay, CAT	Platelet-MPs MP-procoagulant activity	Higher in MPN at diagnosis vs. HDs; higher in thrombotic complications	[101]
MDS	Serum	Ultracentrifugation	FC	CD13	Higher in MDS at diagnosis vs. HDs; higher in high vs. low risk	[15]
			AFM, TEM, qRT-PCR	miR-155	Lower in MDS at diagnosis vs. HDs; monitor MDS progression to AML	[37]
	Plasma	Exosome isolation kit	qRT-PCR	miR-196a-5p, miR-196b-5p, miR-378i	Higher in MDS at diagnosis vs. HDs	[103]
				miR-4267	Lower in MDS at diagnosis vs. HDs	

*MGUS* monoclonal gammopathy of undetermined significance, *SMM* smoldering multiple myeloma, *MM* multiple myeloma, *FC* flow cytometer, *HDs* healthy donors, *DSG* discontinuous sucrose gradient, *AFM* atomic force microscopy, *CGN* colloidal gold nanoplasmonics, *SPR* surface plasmon resonance biosensing, *ISS* international staging system, *DLS* dynamic light scattering analysis, *HPLC* High Performance Liquid Chromatography, *SEM* scanning electron microscopy, *TEM* transmission electron microscopy, *P-gp* P-glycoprotein, *PS* phosphatidylserine, *ELISA* enzyme-linked immunosorbent assay, *GVHD* graft versus host disease, *qRT-PCR* quantitative real-time polymerase chain reaction, *PFS* progression free survival, *OS* overall survival, *HL* Hodgkin’s lymphoma, *NHL* non-Hodgkin lymphoma, *SEC* size exclusion chromatography, *DLBCL* diffuse large B-cell lymphoma, *FL* follicular lymphoma, *CLL* chronic lymphocytic leukemia, *NTA* nanoparticle tracking analysis, *EM* electron microscopy, *WB* western blot, *CM* confocal microscopy, *RS* Richter syndrome, *WM* waldenström macroglobulinemia, *IPSS* International Prognostic Scoring System, *AML* Acute myeloid leukemia, *CT* chemotherapy, *DFS* disease free survival, *CML* chronic myeloid leukemia, *PB* peripheral blood, *TKI* tyrosine kinase inhibitors, *Ph* Philadelphia chromosome, *MPNs* myeloproliferative neoplasms, *MPs* microparticles, *MDS* myelodysplastic syndrome, *CAT* calibrated automated thrombography.

### Lymphoid neoplasms

#### Monoclonal gammopathies

Monoclonal gammopathies, characterized by BM clonal expansion of plasma cells (PCs), comprise a large spectrum of disorders ranging from asymptomatic monoclonal gammopathy of undetermined significance (MGUS) and smoldering multiple myeloma (SMM), to life-threatening diseases, such as multiple myeloma (MM) and amyloid light chain amyloidosis [19]. Determination of the stage, as well as the probable prognosis in MM patients, is a crucial requirement for the most appropriate therapy selection. Indeed, there is an urgent need to identify biomarkers for MM diagnosis, prognosis, monitoring and, even more, for measuring MRD which is responsible for disease relapse and death [19, 41–43].

In this scenario, EVs and their content, could be considered as novel MM biomarkers. Of note, a clinical trial is currently ongoing in Italy to investigate the prognostic role of EVs in elderly MM patients treated with bortezomib-melphalan-prednisone or lenalidomide-desamethasone (MM, Eudract2017-004003-46).

We and others demonstrated that EV count could allow to discriminate between healthy subjects and patients; in addition, it was found that EV number was higher in MM patients with respect to MGUS and healthy subjects [15, 44, 45].

Furthermore, it was widely demonstrated that MM EVs carry specific malignancy markers on their surface, such as CD38 antigen, and that these CD38^+^MVs were significantly more abundant in BM plasma from 27 MM patients at diagnosis compared to 11 MGUS and 14 SMM patients [46]. Likewise, increased levels of CD38^+^MV were detected in MM patient serum and positively correlated with MM clinical International Staging System (ISS) [15]. Similar results were observed for other ectoenzymes which convert adenosine precursors (ATP or NAD^+^) into adenosine in BM niche too, such as CD203a(PC-1), CD73, CD157 and CD39 carried on MM MVs. Moreover, the percentage of MVs expressing high levels of CD203a(PC-1) and CD73 was higher in MM patients at I–II than III ISS stage and the percentage of MVs displaying high expression of either CD38 or CD157 positively correlated with PC percentage [46].

In order to improve EV specificity, many cytofluorimetric studies analyzed the expression of CD138, the gold standard marker to detect MM cells, showing that plasma CD138^+^EVs are higher in MM patients compared to healthy donors, and that their levels are associated with disease phase and therapeutic response [45, 47]. Zhang et al. performed receiver operator characteristic (ROC) curve analysis on peripheral plasma CD38^+^/CD138^+^EV number, distinguishing 61 de novo MM patients from healthy donors, and they also observed a positive correlation between CD138^+^EV count and bone lesion number in MM patients [48]. Moreover, Rajeev Krishnan et al. set a novel blood test in which EVs could be used to monitor disease burden, progression, and development of multidrug-resistance in 74 MM patients (*n* = 14 de novo, *n* = 30 partial remission, *n* = 12 complete remission, and *n* = 18 relapsed). Specifically, they observed that EVs differ in CD138, P-gp, CD34, and phosphatidylserine expression. High levels of P-gp^+^ and phosphatidylserine^+^ EVs positively correlated with disease progression and resistance to treatment. Furthermore, P-gp, phosphatidylserine, and CD34 were mainly expressed in CD138^−^EVs in aggressive/nonresponsive diseases [49]. Interestingly, these data reflect typical CD138 antigen lower expression on MM PCs which is indicative of an immature phenotype, poor prognosis and lower sensitivity to treatment [49]. Although further studies on larger patient cohorts are needed to confirm these findings, this novel test could provide a personalized liquid biopsy with potential to address the unmet clinical need of monitoring multidrug resistance and treatment failure in MM [49].

The substantial outcome of the mentioned studies is the potential use of these surface antigens as minimally invasive and effective systemic biomarkers for MM management.

Since it is known that MM BM is infiltrated by tumor-associated CD163^+^macrophages leading to an unfavorable prognosis [50], Kvorning et al. demonstrated that total concentration of soluble macrophagic antigens, CD163 and CD206, did not vary among 32 newly diagnosed MM patients, 8 MGUS patients and healthy donors; on the contrary, they observed a significantly higher concentration of both CD163^+^ and CD206^+^EVs in plasma of MM patients at diagnosis as compared to remission patients, MGUS and healthy donors. Furthermore, CD163^+^EV level was higher in newly diagnosed MM as compared to relapsed patients. So, macrophage-associated EVs may have monitoring and prognostic biomarker potential in MM [51]. Furthermore, in newly diagnosed (*n* = 5) and relapsed (*n* = 4) MM patients’ follow-up samples, after 2–3 months of treatment, no significant changes in CD163^+^EV and CD206^+^EV levels were observed compared to prior to treatment, while Ecto-CD163 and total sCD163 levels increased significantly during treatment [51]. Although confirmation by survival studies with longer follow-up patients is needed, these data might suggest that Ecto-CD163 reflects acute inflammatory changes in macrophage activation, whereas CD163^+^EV changes are regulated by other mechanisms.

Harshman et al. demonstrated the higher expression of CD44, a glycoprotein implicated in invasiveness, cancer cell trafficking, resistance to apoptosis, and therapy, in circulating EVs of 32 MM patients at diagnosis compared to healthy individuals [52]. Considering that increased levels of soluble CD44 can be associated with MM patient decreased survival, its over-expression on MM EV surface with respect to healthy subjects highlights the considerable role of this antigen as hallmark of cancer EVs.

Exosomes have also been shown to be useful in predicting the risk of graft-versus-host disease (GVHD) following BM allograft transplantation in MM patients. An exploratory study analyzing specific membrane proteins, predictive of acute GVHD, on serum EVs isolated from 41 MM patients, for which serum samples were collected before and after (+28, +58, +92, +119, +147, and +179 days) transplant or at disease relapse, reported that CD146 (melanoma cell adhesion molecule1) expression positively correlated with an increased risk of GVHD, while CD31 (platelet endothelial cell adhesion molecule) and CD140-α (platelet-derived growth factor receptor alpha) expression was indicative of a lower risk of developing this transplant-related complication [53].

EV-derived miRNAs and lncRNAs could also be potentially used as novel biomarkers in MM clinical practice. Manier et al. found 22 miRNAs with significantly lower levels in 156 newly diagnosed MM patients, uniformly treated and followed-up (median follow-up of the cohort was 5.4 years), compared to healthy donors. Among them, they identified downregulation of let-7b, let-7e, miR-106a, miR-106b, miR-155, miR-16, miR-17, miR-18a, and miR-20a as significant predictors for shorter progression-free survival (PFS), whereas downregulation of let-7b and miR-18a as significant predictors for shorter overall survival (OS) [54]. MiR-155 levels were also reported to be significantly lower in a small cohort of MM patients at diagnosis compared to healthy subjects [15]; similarly, serum exosomal let-7c-5p, let-7d-5p, miR-185-5p, miR-20a-5p, miR-103a-3p, miR-425-5p, miR-4741, miR-4505, and miR-140-3p levels were significantly different among 20 MM patients at diagnosis, 20 SMM patients and healthy individuals [55].

Furthermore, Raimondo et al. found that miR-129-5p, which targets different osteoblast differentiation markers, is enriched in BM plasma EVs from MM compared to SMM patients, thus suggesting its correlation with pathological grade [56].

Since the occurrence of MM is often preceded by the asymptomatic SMM [19], a very interesting aspect emerging from MM EV molecular content analysis is that EV miRNAs may represent new biomarkers for risk stratification of SMM patients; therefore, additional studies are needed to elucidate this aspect.

Moreover, early prediction of MM drug resistance through EV miRNAs is one of the most important objectives. Zhang et al. reported the downregulation of exosomal miR-16-5p, miR-15a-5p and miR-20a-5p, miR-17-5p in bortezomib-resistant MM patients, indicating them as potential candidates for a predictive panel of drug resistance biomarkers [57].

Currently, little is known about lnRNAs derived from MM EVs. Sedlarikova et al. analyzed lncRNA expression profiles in serum exosomes from 56 newly diagnosed MM and 49 MGUS patients in comparison with healthy donors, revealing deregulation of exosomal lncRNA PRINS in MM. In addition, ROC curve analysis distinguished MM and MGUS patients from healthy donors, suggesting a possible diagnostic role for exosomal lncRNA PRINS in monoclonal gammopathies patients [58].

#### Lymphoma

Lymphomas are heterogeneous diseases caused by malignant transformation of lymphocytes and affect lymph nodes, BM, and other organs. Hodgkin lymphoma (HL) and non-HL (NHL) are the two main categories of these neoplasms [59]. In addition, NHLs are further divided into several subtypes [60]. Despite a substantial percentage of patients achieves stable remission after chemotherapy, a small percentage presents refractory disease or relapses after treatment and develops chemoresistance [61]. For this reason, the identification of novel prognostic markers and the development of other treatment approaches are an imperative clinical need [61].

In this context, several studies have been performed on circulating EVs as potential biomarkers. Specifically, both number and surface markers of HL- and NHL-derived EVs are related with lymphoma subtypes and correlated with clinical stage [15, 62, 63]. In particular, it has been reported an increased number of serum and plasma EVs in HL and NHL patients compared to healthy subjects [15, 62], and higher level of CD30^+^ and CD19^+^MVs in 11 HL and 10 NHL patients with respect to controls, respectively [15]. Interestingly, the presence of CD30, a typical Reed-Sternberg cell antigen, on HL MVs could render it a specific EV diagnostic marker in this neoplasm [15]. Circulating tumor-derived exosomes may also provide helpful information for conventional anti-cancer immunotherapy. Interestingly, there is only a study, although done on a minimum number of patients after therapy, which showed that plasma B-lymphoma-derived CD20^+^exosomes are able to bind rituximab, an anti-CD20 monoclonal antibody, reducing the number of antibody molecules that can effectively reach tumor cells and decreasing its therapeutic effectiveness [63].

Recently, a large Spanish multicentric study demonstrated the predictive and prognostic role of tumor-associated mRNAs in plasma exosomes of 60 diffuse large B-cell lymphoma (DLBCL) and 38 follicular lymphoma (FL) patients; additional 31 post-treatment samples were also studied. Specifically, authors found higher expression of BCL-6 in exosomes from DLBCL and FL patients than in healthy controls, and BCL-6 and C-MYC mRNAs as predictor markers of shorter OS and worse PFS. Moreover, they investigated the possible role of exosome mRNAs in therapy monitoring and identified an association of BCL-6 levels with the response to rituximab and the risk of death. In addition, high AKT levels resulted associated with worse PFS in therapy responder patients and BCL-XL with a high death rate in FL patients after rituximab treatment [64]. Altogether, these data support the potential role for exosomal RNA as tumor markers to identify high-risk and nonresponder NHL patients.

EV-derived miRNAs could be also used as biomarkers in lymphomas [62, 64–66]. van Eijndhoven et al. collected EVs from plasma of HL patients before, during and after therapy and up to 15 months after treatment initiation. High levels of miR-24-3p, miR-127-3p, miR-21-5p, miR-155-5p, and let-7a-5p were identified in EVs from untreated patients compared with healthy individuals [62]. Moreover, serial monitoring of EV miRNAs in patients, revealed robust, stable decreases in miRNA levels matching a complete metabolic response, as observed with FDG-PET. Importantly, their levels increased again in relapsed patients [62]. These observations not only strongly portray the diagnostic role of these miRNAs but also suggest that their expression levels could be used to monitor treatment response and relapse [62].

Only few studies have analyzed the potential role of exosomal miRNAs as markers of resistance to therapy in lymphoma. Feng et al. found increased levels of miR-99a-5p and miR-125b-5p in exosomes derived from 33 chemoresistant DLBCL patients compared to 83 chemosensitive subjects, suggesting their correlation with shorter PFS time and their possible use to predict therapy efficacy [66].

#### Chronic lymphocytic leukemia

Chronic lymphocytic leukemia (CLL) is a lymphoproliferative disorder characterized by gradual accumulation of morphologically mature B lymphocytes in blood and in primary lymphoid organs. Clinical evolution of CLL is stringently associated with a tumor-supportive microenvironment and with a dysfunctional immune system and is sometimes unpredictable [67].

CLL evolution may manifest through distinct clinical stages that develop from monoclonal B-cell lymphocytosis to CLL and, later, into Richter syndrome [68].

Recently, EVs and their content have been proposed as a useful tool for diagnosis and prognosis of CLL. For instance, several studies reported a higher number of MVs and exosomes in both serum and plasma of newly diagnosed CLL patients compared to healthy subjects [15, 69] and an increased serum MV levels in advanced Rai clinical stages [70]. Of note, ROC analysis of serum MV number from 131 CLL patients, distinguished Rai 0 stage patients with shorter time to treatment from those with more stable disease and, among the entire cohort, patients with shorter OS, thus suggesting serum MV number as a new potential prognostic biomarker in CLL [70]. Interestingly, plasma exosome concentration could be also considered as marker of therapy response due to their significant reduction in 9 patients after Ibrutinib treatment with respect to matched pre-treated ones [69].

It has been demonstrated that EVs express specific CLL antigens whose analysis could have a diagnostic and predictive role in this neoplasm. In particular, a higher number of serum CD19^+^MVs was reported in 11 CLL patients at diagnosis compared to healthy subjects [15], and an increased level of serum CD19^+^, CD20^+^ and CD37^+^MVs was described in advanced CLL compared to early-stage disease [70].

Another study reported a higher number of plasma CD52^+^MVs in 33 untreated CLL patients with respect to healthy subjects and an increased circulation of these MVs compared to CD19^+^ones. In addition, authors explored the possible correlation of plasma CD19^+^ and CD52^+^MV levels with known prognostic risk factors, detecting no significant difference between mutated- vs. unmutated-IGVH status. Moreover, while CD52^+^MVs did not show any association with Rai-risk in CLL patients, their increased levels were reported in high FISH-risk (17p-/11q-) as compared to low FISH-risk (13q-/Tri12 or no genetic abnormalities) patients, thus indicating that further studies are needed to better establish CD52^+^MV role as prognostic marker in this disease. Furthermore, a dynamic change of CD52^+^MV levels was also observed during therapy, suggesting CD52^+^MVs as a possible biomarkers for response to therapy [71].

Concerning protein cargo, proteomic analysis, was carried out on plasma exosomes from two cohorts of CLL patients, respectively, of 5 patients with progressive disease (longitudinally analyzed at diagnosis, during stable disease and at disease progression but before treatment), and 5 patients with indolent disease (evaluated at diagnosis and after 4 years of follow-up without disease evolution). Of note, these data identified S100-A9, a protein promoting inflammation through NF-kB pathway activation, as an exclusive exosome protein of disease progression state, resulting absent or low expressed at diagnosis and in indolent states [72].

Recently, differential expression analysis revealed a significant upregulation of miR-150, miR-155, and miR-29 family members (miR-29a-c) in plasma exosomes of 69 CLL patients at diagnosis as compared with healthy donors, emphasizing their possible diagnostic role in this disease [69]. According to this work, we reported higher levels of miR-155 in serum EVs from 9 CLL patients at diagnosis compared to healthy subjects, and ROC analysis indicated its potential role as diagnostic biomarker in this neoplasm [37].

To date, only a preliminary study identified a plasma exosome miRNA signature that could predict the evolution of therapy-resistant CLL patients towards Richter syndrome [68]. Certainly, this study requires the enrollment of a large cohort of patients to validate exosomal miRNAs as prognostic factors for CLL progression into more aggressive and chemotherapy refractory clinical entity.

#### Waldenström macroglobulinemia

Waldenström macroglobulinemia (WM) is an uncommon lymphoma characterized by BM infiltration with lymphoplasmacytic cells associated with a secretion of IgM proteins [73]. It remains a rare, incurable cancer, with a heterogeneous disease course and progression [73, 74]. There is a need to identify novel biomarkers to better characterize and prevent disease progression from smoldering to symptomatic WM. As for other HMs, EVs could help to identify patients with smoldering/asymptomatic WM at high risk of progression who might benefit from an early therapy.

In this context, we observed that both total and CD19^+^MV count was higher in serum of 12 WM patients at diagnosis compared to healthy controls and that the increased number correlated with high International Prognostic Scoring System (IPSS) compared to those with intermediate and low IPSS [15].

Bouyssou et al. performed miRNA profiling on exosomes isolated from plasma samples of healthy donors and WM patients at progressive stages (30 smoldering/asymptomatic WM and 44 symptomatic WM), and then measured expression levels of selected miRNAs. Of note, they identified four miRNAs whose expression levels correlated with disease progression stages; in particular, onco-miRNAs miR-192-5p, miR-320b, and miR-21-5p increased with the disease stage, whereas tumor suppressor let-7d was downregulated. Thus, these EV miRNAs could be used to track disease progression [75]. Intriguingly, although in-depth studies are required to confirm these data, changes in EV miRNAs can occur before asymptomatic WM progresses into a symptomatic phase suggesting that they could be indicators for an early therapeutic intervention before the development of clinically evident end-organ damage [75].

MiR-155 has been shown to play a critical role in WM pathogenesis, in particular by regulating cell proliferation and growth. WM neoplastic cells exhibit higher miR-155 expression levels than control cells and they positively correlate with the IPSS [76, 77]. Moreover, we showed, for the first time, that miR-155 is present in serum WM EVs and that its level is significantly increased compared to healthy controls. Interestingly, our preliminary data showed a trend for a positive association between high EV miR-155 levels and an intermediate-high IPSS score [37].

### Myeloid neoplasms

#### Acute myeloid leukemia

AML is characterized by clonal proliferation of poorly differentiated cells of myeloid lineage; it is a highly heterogeneous disease and is caused by mutations affecting signaling pathways, as well as transcriptional and epigenetic regulators [78, 79]. In addition, despite considerable progress and a relatively high morphologic remission rate with intensive chemotherapy, most patients relapse due to the presence of MRD [80]. The identification of new AML biomarkers may contribute to a better understanding of the molecular bases of this disease, and may be useful in screening, diagnosis, prognosis, and monitoring of AML, as well as in predicting response to treatment. Due to AML heterogeneity, from a clinical perspective, it would be appropriate to identify a panel of biomarkers able to improve patient classification [81].

Data in literature confirm an increased interest in the field of EVs as novel biomarkers in AML. For instance, it was demonstrated that AML patients at diagnosis display a higher serum EV number than healthy controls. Moreover, these EVs were shown to derive from AML blasts, as indicated by their surface antigens (e.g., CD34, CD117, and CD13) [15, 82]. Therefore, EV derivation from tumor cells is informative about the presence of leukemic blasts in BM and this is important in patient monitoring during therapy [83, 84]. In this context, Hong et al. analyzed the levels of total proteins and transforming growth factor-beta1 (TGFb-1), in plasma EVs from 16 newly diagnosed AML patients and from other patients during chemotherapy (*n* = 9 post induction, *n* = 10 during consolidation, and *n* = 5 long-term remission chemotherapy). Of note, they found an increased level of total proteins and TGFb-1 at diagnosis compared to healthy subjects and that their expression changed during treatment; in particular, a decrease after induction and an increase during consolidation chemotherapy followed by a normalization in long-term chemotherapy were reported. Interestingly, protein levels and TGFb-1 fluctuations during treatment may reflect response to chemotherapy and were related to AML blast presence in BM. Therefore, exosomal proteins and TGFb-1 are proposed as novel potential biomarkers of response to therapy and their level could reflect the presence/absence of residual disease after therapy [83]. However, to strengthen these data, it will be useful to analyze a larger cohort of patients at diagnosis and during treatment and follow-up.

A potential diagnostic power of AML EVs was recently demonstrated by Kunz et al. Specifically, authors detected, in plasma EV RNA isolated from 16 pediatric AML patients at diagnosis, two leukemia-specific mutations, FLT3-ITD and NPM1, reflecting mutational status of matched leukemic blasts. In addition, they found that EV number and RNA amounts appeared to be influenced by the mutational background of patients. However, they performed EV RNA mutational analysis in the same cohort of patients, longitudinally followed after treatment, but the results obtained did not correlate with genomic DNA analysis [84]. This is probably due to the concomitant reduction of leukemic cells and derived-EVs determining the low sensitivity of the performed approach.

A similar study was recently conducted on dsDNA which has been proposed as a noninvasive biomarker in pediatric AML. DsDNA derived from plasma EVs of 20 AML patients at diagnosis and during treatment was examined for leukemia-specific mutations (e.g., NPM1, FLT3, WT1, GATA2, ETV6, ZRSR2, NOTCH1, NRAS, KIT, and PHF6). Similarly to RNA content, authors demonstrated that EV-DNA mirrored the leukemia-specific mutations found in genomic DNA of primary leukemia cells suggesting its utility in AML patients at diagnosis [85]. However, both EV-DNA and genomic DNA showed the absence of AML specific mutations or SNPs in patients after treatment [85], probably for the same limitations mentioned for RNA mutational analysis.

EV miRNAs were also suggested as potential biomarkers in AML [37, 81, 86–88]. Analysis of miR-155, a miRNA deregulated in this neoplasm [89], showed its significant higher level in serum MVs from 11 AML patients at diagnosis compared to healthy subjects; moreover, ROC curve analysis revealed that miR-155 could be a new potential diagnostic biomarker in AML [37]. Since this cellular miRNA is involved in processes altered in AML, such as proliferation and myeloid differentiation, studies evaluating EV miR-155 expression in a large cohort of patients could elucidate its possible clinical relevance in this neoplasm.

Likewise to miR-155, Fang et al. analyzed serum EV miR-10b level, a miRNA previously described as upregulated in AML cells, reporting its higher expression in 95 de novo AML patients compared to healthy volunteers. In AML it has been reported the existence of distinct miRNA profiles in different disease subtypes, indicating miRNA signature contribution to AML heterogeneity, and suggesting its potential inclusion in clinical setting [90]. Interestingly, EV miR-10b levels were significantly higher in all AML subtypes based on both French-American-British and World Health Organization classifications. So, these data suggest that EV miR-10b alteration is not associated to a specific AML subtype. However, cellular miR-10b was found upregulated in patients harboring NPM1 mutation [86], thus it could be interesting to evaluate it in patients with this specific genetic abnormality. In addition, ROC curve on EV miR-10b levels yielded a good diagnostic power in discriminating AML cases from normal controls. Serum EV miR-10b was also closely associated with poor prognosis, being more highly expressed in AML patients with shorter OS and disease-free survival, and resulting an independent prognostic factor for OS [86].

Recently, Jang et al. investigated the prognostic role of circulating miR-125b, an oncogenic miRNA, in a cohort of 154 AML patients with intermediate-risk. In particular, they found that exosomal miR-125b was higher in patients at diagnosis with respect to healthy subjects. Moreover, miR-125b increased levels correlated with higher risks of relapse and overall death, supporting its role as an independent prognostic indicator in this intermediate-risk group of patients [87].

In a murine model of AML, a panel of miRNAs isolated from serum EVs have been suggested as minimally invasive early biomarkers. Specifically, authors developed a biostatistical model, by a miRNA scoring algorithm, able to discriminate leukemia-engrafted mice from controls and to detect circulating exosomal miRNAs at low marrow tumor burden and before detection of circulating blasts. This score was also applied for miR-150, miR-155, and miR-1246 isolated from circulating EVs of a small preliminary cohort of patients identifying a cut-off able to discriminate patients from normal subjects, thus suggesting these miRNAs as new potential biomarkers in AML [81]. Notably, this study provided a platform for the development of clinical AML biomarkers and corroborated the concept that serum EV miRNAs can add sensitivity and specificity to minimally invasive detection of residual or recurrent AML, conferring further support to their suitability as cell-free biomarkers unaffected by chemotherapy.

#### Myeloproliferative neoplasms

CML, a clonal myeloproliferative neoplasm carrying the Philadelphia chromosome (Ph), is characterized by an initial chronic phase, an intermediate accelerated phase and a final, fatal, blastic phase [91, 92]. Numerous studies have demonstrated the persistence of CML leukemic cells in the BM niche following treatment, even in patients with undetectable levels of the BCR-ABL1 transcript, proving that the standardized MRD monitoring system is not always effective [32, 93]. Indeed, these findings make CML a suitable model to investigate new possibilities for the detection of residual tumor*-*cell activity by exosome analysis [32].

Recently, it was found that both total and myeloid CD13^+^MV are higher in serum of CML patients at diagnosis than in healthy donors [15]. Furthermore, different studies demonstrated the presence of the BCR-ABL1 transcript in EVs and its potential role as biomarker in CML patients [24, 32]. In particular, a preliminary study, carried out on samples at different time points, reported high serum exosomal BCR-ABL1 mRNA levels in patients at blast or accelerated phase and not in chronic phase, and their decrease in response to TKI treatment [24]. Another study revealed BCR-ABL1 presence in plasma exosome from 10 CML patients already in chronic phase [32]. Interestingly, the applied leukemia-exosome enrichment method combined with highly sensitive digital PCR quantification could explain BCR-ABL1 detection in an initial disease phase.

Thus, further studies are needed to evaluate whether EV BCR-ABL1 mRNA could be helpful in differentiating chronic from advanced phases of disease and may be a more objective indicator for the accurate identification of CML phases than the available ones [94].

Other authors correlated the expression of exosomal miRNAs from PB of CML patients with musculoskeletal pain after stopping TKIs, to identify possible factors related to this clinical manifestation. Specifically, miRNA profiling revealed that exosomal miR-140-3p was significantly elevated in CML patients affected by musculoskeletal pain, when compared to those without such pain or healthy individuals, thus resulting as a possible biomarker of this complication [95].

Among myeloproliferative neoplasms (MPNs), due to their overlapping features, polycythemia vera, essential thrombocythemia (ET), and primary myelofibrosis have been traditionally grouped into a unique category of Ph^−^ classical MPNs [94]. One of the main problems of MPN patients is the high risk and incidence of thrombosis which affects survival, quality of life, and life expectancy [96].

Several reports indicate the increased release of blood MPs in these neoplasms. In particular, MPs derived from different cell sources, such as platelet-, erythrocyte-, red blood cell-, and endothelial-MPs, were found significantly augmented in MPN patients compared with healthy subjects. Interestingly, blood MP levels are positively correlated with the occurrence of thrombotic complications in MPN patients [96–98]. In addition, another recent study analyzed plasma levels of tissue factor positive-MPs from 59 MPN patients at diagnosis and during clinical course of disease, and showed that their procoagulant activity was significantly higher in patients suffering thrombotic events than in patients without such events. In addition, authors determined, by ROC analysis, an MP cut-off value showing that tissue factor positive MP levels correlated with patient thrombotic history [99]. Since the prevention of thrombotic events is a primary aim of current treatment for MPN disorders, understanding MP role as biomarkers of this event could be useful in the management of these diseases.

Given the numerous observations suggesting MP contribution in ET pathophysiology, recently, Piccin et al. conducted a retrospective study to assess the potential relationship between MP release and endothelial modulators. In particular, in a cohort of 63 patients, analyzed at diagnosis and longitudinally during therapy, treatment with drugs reducing platelet count affected MP generation by altering endothelial modulator production. These findings underline MP potential role in the clinical course of ET disease [100].

Finally, plasma MPs were analyzed in 72 ET patients by Charpentier et al. Specifically, authors found that ET patients harboring JAK2-V617F mutation, at diagnosis, showed more circulating platelet derived-MPs and a higher MP-associated procoagulant activity than CALR-mutated and triple-negative ET patients. Thus, platelet-MP might contribute to a higher incidence of thrombosis in ET patients and, at least in part, to the distinct thrombotic risk according to their mutational status [101].

#### Myelodysplastic syndrome

Myelodysplastic syndromes (MDS) are a heterogeneous group of clonal stem cell disorders, characterized by manifestations of BM failure causing an ineffective hematopoiesis, cytopenia, single- or multi-lineage dysplasia, and an inherent tendency to leukemic transformation [102]. Outcome for MDS patients is heterogeneous, then individual risk stratification is important in managing patients. The identification of novel biomarkers may allow a better evaluation of the disease and improve prognosis, thus helping clinicians in the decision-making process [102].

To date, little is known about circulating EVs in MDS. The first evidence revealed the expression of surface myeloid marker CD13 on serum MVs derived from 5 MDS patients with respect to healthy subjects. Interestingly, higher risk MDS displayed an increased level of serum CD13^+^MVs [15].

Furthermore, MV miR-155 levels were significantly lower in 5 MDS patients at diagnosis compared to healthy subjects. It was also found that miR-155 levels were higher in very high R-IPSS score with respect to low ones [37]. From a clinical point of view, it is well known that low-risk MDS patients can progress to high-risk and that, these last patients, could progress to AML. Likewise, EV miR-155 levels progressively increased from low to high-risk MDS to AML patients in which, as reported in the previous section, miR-155 resulted upregulated compared to healthy subjects. Of note, ROC curve analyses of serum MV miR-155 levels discriminated between MDS and AML patients suggesting that this miRNA may be used to monitor MDS progression [37]. Thus, it could be interesting to evaluate EV miR-155 expression in a larger number of MDS patients with different risk, at diagnosis and longitudinally followed, to include patients with secondary AML. In this way it could be possible to obtain a cut-off potentially discriminating from MDS at low to high risk ones evolving in AML.

Another recent study analyzed an extensive panel of 372 plasma exosomal miRNAs in a discovery cohort of MDS patients at diagnosis and healthy controls. Among differentially expressed miRNAs between these two groups, they found miR-196a-5p, miR-196b-5p, miR-378i upregulated, and miR-4267 downregulated in MDS validation cohort. Of note, ROC curve analysis assessed these exosomal miRNAs as potential new diagnostic biomarkers in MDS [103].

## Conclusions

Cancer is a serious health issue, being one of the leading causes of death worldwide. The survival rate of patients remains unsatisfactory due to the late diagnosis, frequent relapse, and poor response to therapy. Therefore, novel methods with high specificity and sensitivity for early cancer detection and monitoring are needed to select the most appropriate treatment and for a deeper evaluation of MRD.

**Fig. 2:**
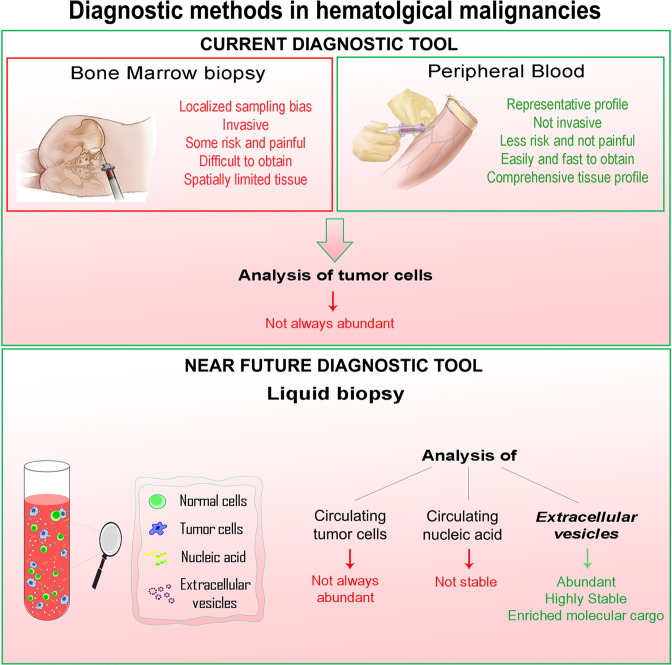
Advantages and disadvantages of current and near future diagnostic tools in hematological malignancies. Diagnostics of hematological malignancies is currently based on bone marrow (BM) and peripheral blood (PB) analyses. BM biopsy presents several disadvantages (in red), such as localized sampling bias, invasiveness, risk and pain, and spatial limitation. PB analysis overcomes BM limitations, being tumor representative, noninvasive, easily and fast to obtain. Sampling of both BM and PB sources allow the analysis of tumor cells that are not always abundant. Liquid biopsy represents a near future diagnostic tool and includes analysis of circulating tumor cells, nucleic acids and/or EVs. These last analytes are the most abundant and stable in PB and are characterized by an enriched molecular cargo which can be considered representative of the cells of origin.

EV-based liquid biopsy provides a noninvasive, fast, pain- and hassle-free diagnostic method, alternative to BM biopsies, for the detection and monitoring of hematological cancers (Fig. 2).

EVs derived from serum/plasma of HM patients contain a complex cargo (proteins, mRNAs, miRNAs, etc.) which might represent a snapshot of the disease state, being real representative of tumor. EV characteristics (amount, phenotype, and content) are able to provide multiple information serving as novel and promising biomarkers in HMs. In addition, circulating EVs have a great potential to refine current diagnostic and prognostic criteria and can be used as a novel strategy to monitor dynamic changes during disease development and therapy in HMs.

In order to translate these novel findings into clinical practice, several questions remain open. The main issue concerns robustness and reproducibility of data in the entire procedure, from EV isolation to detection and analysis, up to the investigation of their content. Therefore, it is necessary to set up standardized approaches for EV assessment and their clinical applicability. Planning and conducting consortium-type studies involving multiple laboratories will accelerate progress toward standardizing experimental approaches and for data analysis. In addition, findings deriving from preclinical studies might help to further improve clinical trials. Notably, a considerable number of trials, registered on www.ClinicalTrials.gov database, are already underway in solid tumors, and this makes concrete the EV use in clinics.

Furthermore, rapid development of novel technologies can improve EV biomedical and clinical applications. In particular, different emerging methods, such as raman spectroscopy and frequency-locked optical whispering evanescent resonance, allow EV identification and their quick measurement, and could have the potential to identify the origin of a specific vesicle [14]. Moreover, mass spectrometry, as above mentioned, and SOMA-scan, an affinity-based proteomic analysis technique, are used for highly sensitive and specific protein detection in human blood and other biomatrices possibly finding novel protein biomarker candidates [14]. In addition, immune-droplet digital PCR amplification method allows multiplex protein analysis in single EV [104].

Future advancements in this field will certainly lead to the development of new methodologies for fast and reliable EV characterization assays at low costs. Moreover, thanks to the development of sophisticated and miniaturized “omics” approaches and modern technologies, such as microarray profiling, digital PCR arrays, and NGS, it will be possible to detect specific EV signatures.

In conclusion, the reports mentioned in this review highlight the potential suitability of EVs as novel analytes that can be translated, in the near future, in clinical practice possibly favoring a better management of hematological neoplasms.
